# Investigation of occupational and environmental causes of respiratory cancers (ICARE): a multicenter, population-based case-control study in France

**DOI:** 10.1186/1471-2458-11-928

**Published:** 2011-12-14

**Authors:** Danièle Luce, Isabelle Stücker, ICARE study group

**Affiliations:** 1 INSERM UMRS 1018, CESP, Epidemiology of occupational and social determinants of health Centre for research in Epidemiology and Population Health, 15/16, avenue Paul Vaillant Couturier, 94807 Villejuif, France; 2 UMRS 1018, University of Versailles Saint-Quentin, Villejuif, France; 3 INSERM UMRS 1018, Environmental epidemiology of cancer, Centre for research in Epidemiology and Population Health, Villejuif, France; 4 UMRS 1018, University Paris Sud, Villejuif, France

## Abstract

**Background:**

Occupational causes of respiratory cancers need to be further investigated: the role of occupational exposures in the aetiology of head and neck cancers remains largely unknown, and there are still substantial uncertainties for a number of suspected lung carcinogens. The main objective of the study is to examine occupational risk factors for lung and head and neck cancers.

**Methods/design:**

ICARE is a multi-center, population-based case-control study, which included a group of 2926 lung cancer cases, a group of 2415 head and neck cancer cases, and a common control group of 3555 subjects. Incident cases were identified in collaboration with cancer registries, in 10 geographical areas. The control group was a random sample of the population of these areas, with a distribution by sex and age comparable to that of the cases, and a distribution by socioeconomic status comparable to that of the population. Subjects were interviewed face to face, using a standardized questionnaire collecting particularly information on tobacco and alcohol consumption, residential history and a detailed description of occupational history. Biological samples were also collected from study subjects. The main occupational exposures of interest are asbestos, man-made mineral fibers, formaldehyde, polycyclic aromatic hydrocarbons, chromium and nickel compounds, arsenic, wood dust, textile dust, solvents, strong acids, cutting fluids, silica, diesel fumes, welding fumes. The complete list of exposures of interest includes more than 60 substances. Occupational exposure assessment will use several complementary methods: case-by-case evaluation of exposure by experts; development and use of algorithms to assess exposure from the questionnaires; application of job-exposure matrices.

**Discussion:**

The large number of subjects should allow to uncover exposures associated with moderate increase in risks, and to evaluate risks associated with infrequent or widely dispersed exposures. It will be possible to study joint effects of exposure to different occupational risk factors, to examine the interactions between occupational exposures, tobacco smoking, alcohol drinking, and genetic risk factors, and to estimate the proportion of respiratory cancers attributable to occupational exposures in France. In addition, information on many non-occupational risk factors is available, and the study will provide an excellent framework for numerous studies in various fields.

## Background

Occupational studies are particularly useful for identifying new carcinogens because exposures in the workplace are of higher intensity and more identifiable than in other environments. As a consequence, many of the established carcinogens were first discovered in occupational settings. Occupational exposures also have some distinctive features, as compared to other risk factors for cancer such as diet or smoking. These exposures are mostly involuntary and affected workers are often unaware of them. On the other hand, cancer risks associated with these exposures could be prevented relatively easily, by eliminating the most hazardous substances and by reducing exposure levels, using improved collective and individual protection devices and operational processes.

Obviously, some occupational carcinogens have not yet been identified or evaluated. More than 100 occupational exposures are established animal carcinogens for which human evidence is inadequate or not available. Excess risks of cancer have also been found in a number of occupations and industries for which the specific agents have not been identified [1]. Studies are needed to assess these suspected carcinogens, and to identify the hazardous agents. Almost all cancer types are concerned but respiratory tract cancers are the most frequent, since many substances present in occupational settings are inhaled.

The lung is the target organ for many substances, compounds or exposure settings that are known human carcinogens, among them asbestos, crystalline silica, polycyclic aromatic hydrocarbons, various metals (arsenic, cadmium, beryllium, some nickel and chromium compounds) and chemicals (bis-chloromethylether). A number of other substances or complex compounds are probable or possible lung carcinogens, for example, welding fumes, lead, vinyl chloride, non arsenical insecticides [1,2]. Elevated lung cancer risks have also been found in several occupations or industries, particularly among painters [2] and, less consistently, in the rubber industry [3], among butchers [4], hairdressers [5], woodworkers [6], leather workers [7] and printers [8,9].

The role of occupational exposures in the aetiology of head and neck cancers remains largely unknown. Exposures to strong inorganic acids [10] and to asbestos [11] increase the risk of laryngeal cancer. Several other occupational exposures, notably lung carcinogens, are plausible risk factors for head and neck cancer. Associations between head and neck cancer and exposure to formaldehyde [12], coal dust [13,14], welding fumes [15], cement dust [16], silica [17] and cutting fluids [18] have been suggested. Elevated risks of head and neck cancer have been reported among drivers [19,20], painters [21,22], butchers [4,23], construction workers [23,24], textile workers [20,24] and dry cleaning workers [25-27]. However, none of these associations are firmly established.

Besides identifying new carcinogens, the effects of multiple occupational exposures, the evaluation of interactions between occupational exposures and life-style risk factors such as smoking or alcohol drinking are areas needing work. Similarly, few studies have examined interactions between genetic factors and occupational exposures, and they lack statistical power.

The population-based case-control design is appropriate for these topics, because it is possible to collect information on an extensive range of risk factors, and it is generally easier and less expensive to collect biological samples in this context. In addition, population-based studies allow estimating attributable risks. However a large number of subjects are needed to achieve sufficient statistical power to be able to study interactions, or to identify unknown carcinogens that are probably associated with moderate elevation in risk.

To address these issues, we set up a large population-based case-control study, ICARE (Investigation of occupational and environmental CAuses of REspiratory cancers), which includes a group of lung cancer cases, a group of head and neck cancer cases, and a common control group for all cancer sites.

The objectives are:

- to investigate occupational risk factors for lung and head and neck cancers, and to identify new occupational carcinogens;

- to study joint effects of exposure to different occupational risk factors;

- to examine the interactions between occupational exposures and the major risk factors for the cancer sites of interest (tobacco smoking and alcohol drinking);

- to study interactions between genetic risk factors and occupational carcinogens

In this paper we describe the study design, the methods, the main characteristics of the study subjects and the current progress, as well as the perspectives for further developments.

## Methods/design

### Study design

**Figure 1:**
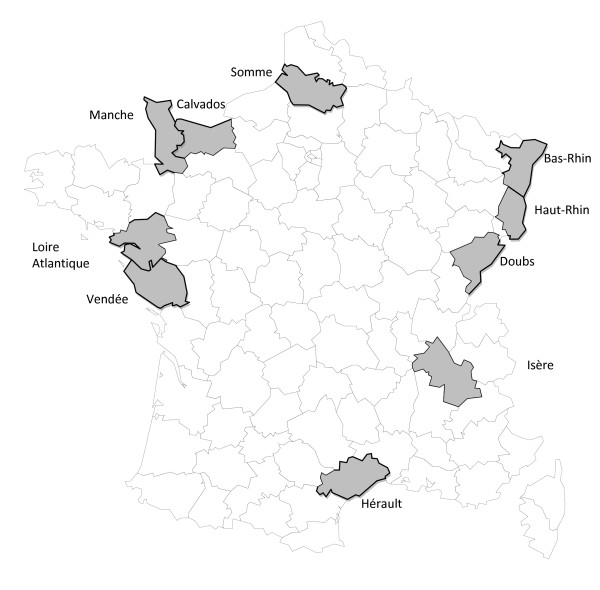
**Geographical area covered by the ICARE study (10 "départements")**.

ICARE is a multi-center, population-based case-control study, which included a group of lung cancer cases, a group of head and neck cancer cases, and a common control group. Incident cases were identified in collaboration with the French cancer registries. Ten general cancer registries, out of the 11 registries operating at the time of study implementation, agreed to collaborate. These registries cover 10 French "départements" (Figure 1), which overall gather about 13% of the French population (7.6 million inhabitants). The distribution of the main occupational and economic activity characteristics of the active population of these regions is similar to their distribution in France (Table 1). The control group was a random sample of the population of these areas.

**Table 1:** Distribution of the active population by socioeconomic status and economic activity, in whole France and in the geographical area covered by the ICARE study

	France (%)	Geographical area covered by ICARE (%)
**Socioeconomic status**		

Farmers	2.7	2.8

Self-employed	6.6	6.4

Managers/professionals	13.1	11.2

Intermediate occupations	23.1	22.7

Clerical, sales and service workers	28.8	27.6

Blue collar workers	25.6	29.2

**Economic activity**		

Agriculture, hunting and forestry	4.0	3.9

Fishing	0.1	0.3

Mining and quarrying	0.2	0.2

Manufacturing	17.3	20.8

Electricity, gas, and water supply	0.9	0.8

Construction	5.8	6.2

Wholesale and retail trade; repair of motor vehicles and personal and household goods	13.2	13.3

Hotels and restaurants	3.5	3.5

Transport, storage and communication	6.4	5.6

Financial intermediation	3.0	2.3

Real estate, renting and business activities	11.2	10.0

Public administration	9.8	8.8

Education	7.3	7.4

Health and social work	11.6	11.9

Other service activities	4.4	3.9

Private households with employed persons	1.1	1.0

Extra-territorial organizations	0.1	0.1

### Cases selection

Cases were all patients residing in the designated "départements" newly diagnosed with a primary, malignant tumor of the oral cavity, pharynx, sinonasal cavities, larynx, bronchi and lung (International Classification of Diseases, 10th Revision, codes C00-C14; C30-C34), during the study period which varied according to study center (see Table 2). Only histologically confirmed cases aged 75 or less at diagnosis were eligible. All histological types were included.

**Table 2:** Distribution of cases and controls by study center

	Lung cancer cases			Head and neck cancer cases			Controls		
**Study center**	**N**	**%**	**Period of diagnosis**	**N**	**%**	**Period of diagnosis**	**N**	**%**	**Recruitment period**

Bas Rhin	398	13.6	06.2004-09.2006	261	10.8	06.2004-06.2007	469	13.2	07.2004-09.2007

Calvados	354	12.1	03.2003-09.2006	247	10.2	03.2003-06.2007	462	13.0	04.2003-09.2007

Doubs	149	5.1	05.2004-06.2006	51	2.1	05.2004-06.2006	143	4.0	06.2004-09.2006

Haut Rhin	73	2.5	11.2004-09.2006	45	1.9	11.2004-10.2006	118	3.3	12.2004-01.2007

Hérault	334	11.4	08.2001-01.2006	256	10.6	08.2001-10.2006	450	12.7	09.2001-01.2007

Isère	476	16.3	08.2001-01.2006	288	11.9	08.2001-10.2006	501	14.1	09.2001-01.2007

Loire Atlantique	350	12.0	08.2004-09.2006	389	16.1	08.2004-06.2007	404	11.4	09.2004-09.2007

Manche	320	10.9	03.2003-09.2006	264	10.9	03.2003-06.2007	312	8.8	04.2003-09.2007

Somme	321	11.0	04.2002-06.2006	430	17.8	04.2002-10.2006	499	14.0	05.2002-01.2007

Vendée	151	5.2	08.2004-09.2006	184	7.6	08.2004-06.2007	197	5.5	09.2004-09.2007

**Total**	2926	100.0		2415	100.0		3555	100.0	

Poor survival for some of the cancers included in this study did not allow to rely on routine inclusion of cases in the registries for their identification. A procedure was set up to expedite case identification, in order to reduce the delay between diagnosis and interview of cases. Cases were identified from pathology laboratories, which are the first to know of a case's diagnosis, and from hospital departments. A list of laboratories and hospital departments that had reported lung and head and neck cancer cases in the two previous years was established by each registry. Interviewers contacted these laboratories and hospitals regularly to collect names and addresses of eligible patients and their physicians.

The interviewers then contacted the physician and requested permission to include their patients in the study. A letter was then sent to the patients' home, or handed to them at the hospital if they were hospitalized, informing them about the study and requesting their participation. If the patients agreed, direct contact was then made with them to arrange an interview and to collect written consent.

### Controls selection

Controls were selected by list-assisted random digit dialing sampling, in the same "départements" as the cases, using incidence density sampling method. Recruitment of controls was done by telephone by a polling institute experienced in this type of procedure and possessing the necessary tools and personnel. First, a random sample of listed numbers was drawn from telephone directories. A list of random numbers was then obtained by adding 1 to the last number, in order to reach unlisted numbers.

Telephone calls were generated automatically by a specifically designed system that allows to define the number of calls to a phone number, given the results of previous calls, and manages the days and time on which the calls should be made. Each number was called 10 times before being abandoned as not answered. Calls were made in the evening on weekdays and during the day on Saturdays, so that each home had the same probability of being contacted. Telephone recruiting was done by trained interviewers from the polling institute. These interviewers had followed a half-day specific training session to better understand the objectives of the study, so that they could better answer eventual queries from the contacted subjects.

When an eligible subject was contacted at home, the objectives of the study and terms of participation (notably duration of interview, donation of buccal cells) were explained and agreement to participate was sought. If contacted persons agreed to participate, they were informed that the ICARE study interviewer would contact them soon, and an information letter was sent.

Controls were frequency-matched to the cases by sex, age (in 4 categories: less than 40, 40-54, 55-64, >65) and residence area ("department"). The distribution by sex and age among the controls was initially based on that of all cases (lung and head and neck cancers) notified to the registries in the two previous years, and was later adjusted as necessary depending on the age and sex distributions of cases and controls recruited at that time. Additional stratification was used to achieve a distribution by socioeconomic status among the controls comparable to that of the general population. The distribution by socioeconomic status of the population in each sex-age-residence group was obtained from census data and from the surveys on employment by the French National Institute of Statistics (INSEE) [28]. Seven categories of socioeconomic status were used, according to the standard French classification [29]: farmers; self-employed workers; managers/professionals; intermediate occupations; clerical, sales and service workers; blue-collar workers; inactive. Unemployed jobseekers or retired workers were classified according to the category of their last job held.

Control recruitment waves were conducted approximately every 2 months. The number of recruits required was based on the estimated number of cases and the expected participation rate among controls, in order to obtain a 1:1 control to case ratio for each group of cases (lung or head and neck). Every 2 months, a list of potential controls was given in each center to the interviewers, who contacted them in turn, to confirm their agreement to participate and to make an appointment for the interview.

### Epidemiological data collection

Subjects were interviewed face to face, using a standardized questionnaire collecting the following information:

- Socio-demographic characteristics (sex, age, marital status, educational level, occupation of parents and spouse, country of birth and parents' country of birth);

- Residential history (successive addresses of residency since birth, with information on type of dwelling and period of construction);

- Anthropometric characteristics (height, weight at interview, weight 2 years before interview, weight at 30 years of age);

- History of cancer and various other diseases;

- History of cancer among first-degree relatives;

- Smoking of cigarettes, cigars, cigarillos and pipe, with beginning and end dates, quantity per day, type of cigarette (blond or brown tobacco, filtered or not, brand), for each smoking episode; questions on snuff or chewing tobacco;

- Passive smoking during childhood, at workplace and at home during adulthood;

- Alcohol consumption, with beginning and end dates, quantities, type of alcohol (wine, beer, cider, 20° "apéritifs", liquor) for each period of regular consumption;

- Detailed occupational history

The occupational history questionnaire was designed with the collaboration of industrial hygienists. It includes general questions on occupational history with, for each job held, information on the company production and activities, a description of tasks performed (nature, frequency, material used) and a description of the work environment. In addition, specific questionnaires were designed concerning tasks or occupations frequently encountered or of special interest for this study, in order to help the interviewer for more technical questioning. These specific questionnaires cover the following tasks, occupations or industry branch: painting, welding/gas cutting/soldering, insulation, tool makers and machinists, foundry workers, furnacemen and coke production workers, hairdressers, tannery and leather workers, wood workers, printing, agriculture, glass industry, miners and quarrymen, rubber industry, chemical industry, construction workers, garage workers/motor vehicle mechanics, plumbers and pipefitters, textile industry.

When subjects were too sick to answer the complete questionnaire interviewers used a summary version of the questionnaire, either for in-person interviews or to interview a next-of-kin. This shorter version included mainly information on smoking, alcohol drinking and occupational history.

### Clinical data collection

In the cancer registries, diagnoses were verified and topography and morphology of the tumors were coded according to the International Classification of Diseases for Oncology, 3rd edition (ICD-O-3), using standardized procedures that follow international guidelines [30].

The specific identification number used for each subject in the ICARE study was added to the case record in the registry database. This gives the possibility to subsequently retrieve additional clinical data, information on vital status or to collect tumor samples.

### Biospecimen collection

Buccal cell collection was used to obtain germline DNA. This method is simple, non-invasive, well accepted by the subjects and efficient for a large population-based study. Buccal cells were collected by the subjects themselves under instruction from the interviewer, by scraping the cheeks with cytobrushes, using 4 cytobrushes by subject (2 for each cheek).

After collection, cytobrushes were placed into 4 independent tubes and sent the same day for storing at -80°C to the Epigenetec Biological Resources Center.

### Data management and quality control

The interviewers received specific training, mainly on the topic of the occupational questionnaire. After the initial 2-day training session, regular meetings were held with interviewers (every 3 or 4 months). These meetings helped maintain interviewers' motivation and level of training. The interviewers also had frequent contacts with the investigating team (verification of inclusion criteria, additional information requests).

The completeness of questionnaires and the quality of interviews were routinely checked, before and during data entry (by automatic control of admissible values and internal consistency). All questionnaires were scanned in order to facilitate storage and possible return to the questionnaire.

All jobs held during the working life were coded, according to the International Standard Classification of Occupations (ISCO) for occupations [31] and to the French Nomenclature of Activities (NAF) for industries [32]. Coders received special training to ensure standardized coding. A handbook used during the training and given to coders was developed. This handbook was subject to regular additions based on problems encountered by the coders. The homogeneity of the coding was checked regularly with a feedback to the coders when necessary. Coders were blind as to case/control status. More than 44000 jobs have been coded, on average 4.9 jobs per subject.

Following completion of data collection, we created a secure collaborative workspace for investigators involved in ICARE data analyses, with access to a central relational database, scans of the questionnaires, data dictionary, meeting minutes, and various documents. A website is currently being developed.

### Occupational exposures assessment

The case-control study design requires retrospective assessment of occupational exposures. The main occupational exposures of interest are asbestos, man-made mineral fibers, formaldehyde, polycyclic aromatic hydrocarbons, some chromium and nickel compounds, arsenic, wood dust, textile dust, solvents, strong acids, cutting fluids, silica, diesel fumes and welding fumes. The complete list of exposures of interest includes more than 60 substances. Exposure assessment is conducted in collaboration with industrial hygienists. Several complementary methods are used: a case-by-case evaluation of exposure by experts, using detailed description of work tasks; development and use of algorithms to assess exposure from the questionnaire responses; application of job-exposure matrices. It is planned to use the job-exposure matrices being developed at the Department of Occupational Health of the "Institut de Veille Sanitaire" (National Institute for Public Health Surveillance) [33]. Several job-exposure matrices are already available or will be available soon: flour dust, leather dust, cement dust, petroleum solvents, chlorinated solvents, silica, mineral wools, refractory ceramic fibers, asbestos, wood dust, polycyclic aromatic hydrocarbons, formaldehyde.

A combination of methods will be used to assess most exposures. Job exposure matrices and/or algorithms will provide a first evaluation of exposure. These initial assessments will be further refined by an individual assessment by experts. The assessment of exposure to asbestos, man-made mineral fibers and solvents is in progress.

### Ethical aspects

Each subject gave a written and informed consent. In order to protect the confidentiality of personal data and to fulfill legal requirements, the questionnaire included only an identification number, without any nominative information. The same identification number was used for biological specimen. The link between the name and the identification number (to the exclusion of any other data) was kept by the cancer registry of the area where the subject was interviewed.

The study was approved by the Institutional Review Board of the French National Institute of Health and Medical Research (IRB-Inserm, n° 01-036), and by the French Data Protection Authority (CNIL n° 90120).

### Descriptive data

Recruitment was conducted between 2001 and 2007. We initially recruited 5926 potentially eligible lung cancer cases. Among them, after diagnosis review in the cancer registries, 1061 were found not to meet the inclusion criteria. Among the 4865 eligible lung cancer cases identified, 486 (10.0%) could not be located, 781 (16.1%) deceased before the interview, 238 (4.9%) could not be interviewed because of poor health. Among the 3360 contacted cases, 2926 (87.1%) agreed to participate.

Among the 5134 potentially eligible head and neck cancer cases recruited, 1087 did not meet the inclusion criteria after diagnosis review. Among the 4047 eligible head and neck cancer cases, 596 (14.7%) could not be located, 299 (7.4%) deceased before the interview, 225 (5.6%) could not be interviewed because of poor health. Among the 2927 contacted cases, 2415 (82.5%) agreed to participate.

A total of 2926 lung cancer cases and 2415 head and neck cancer cases were interviewed, on average within 3 months of diagnosis. Among them, 242 (8.3%) lung cancer cases and 257 (10.6%) head and neck cancer cases completed the interview with the shorter version of the questionnaire. Among the 4673 controls initially recruited, 230 (4.9%) could not be reached by the interviewer, 5 (0.1%) deceased before the interview, 27 (0.6%) could not be interviewed because of poor health. Among the 4411 controls who could be contacted, 3555 (80.6%) agreed to participate and completed the interview. The shorter version of the questionnaire was used for 74 (2.1%) controls.

Table 2 shows the recruitment period and the distribution of cases and controls included in the study according to study center.

Among the 2926 lung cancer cases, 2415 head and neck cancer cases and 3555 controls with interview data, buccal cells were collected for respectively 2233 (76.3%), 1774 (73.5%) and 2931 (82.4%). For now, DNA has been extracted from one cytobrush per subject. Mean quantity of DNA per cytobrush is 800 ng.

The distribution of cases and controls by sex and age is shown in Table 3. As expected, the majority of cases are men, and less than 20% were diagnosed before the age of 50.

**Table 3:** Distribution of cases and controls by sex and age

	Lung cancer cases		Head and neck cancer cases		Controls	
	
	N	%	N	%	N	%
Sex						

Men	2276	77.8	2054	85.1	2780	78.2

Women	650	22.2	361	14.9	775	21.8

Age						

<40	60	2.1	50	2.1	116	3.3

40-45	121	4.1	101	4.2	352	9.9

45-49	290	9.9	313	13.0	361	10.2

50-54	446	15.2	454	18.8	363	10.2

55-59	552	18.9	538	22.3	653	18.4

60-64	507	17.3	406	16.8	535	15.0

65-69	486	16.6	280	11.6	651	18.3

≥70	464	15.9	273	11.3	524	14.7

Total	2926	100.0	2415	100.0	3555	100.0

Table 4 presents the distribution of lung cancer cases by histologic type and by sex. The proportion of adenocarcinomas is higher in women than in men, whereas the proportion of squamous cell carcinomas is lower.

**Table 4:** Distribution of lung cancer cases by histologic type and sex

	Men		Women		All	
**Histologic type**	**N**	**%**	**N**	**%**	**N**	**%**

Squamous cell carcinoma	874	37.0	112	16.9	986	32.6

Small cell carcinoma	335	14.2	96	14.5	431	14.3

Adenocarcinoma	800	33.9	355	53.7	1155	38.2

Large cell carcinoma	199	8.4	46	7.0	245	8.1

Other	153	6.5	52	7.9	205	6.8

More than 90% of head and neck cancer cases are squamous cell carcinomas (2195 out of 2415). The distribution of head and neck cancer cases by subsite is shown in Table 5. Overall, there were 797 cases with oral cavity cancer, 487 with oropharyngeal cancer, 432 with hypopharyngeal cancer, and 529 with laryngeal cancer. The distribution by cancer site in women differs from that among men, cancers of the oral cavity being relatively more common in women, while laryngeal and hypopharyngeal cancers are more frequent in men.

**Table 5:** Distribution of head and neck cancer cases by subsite and sex

		Men		Women		All	
**Subsite**	**ICD-O-3 code**	**N**	**%**	**N**	**%**	**N**	**%**

Oral cavity	C00.0-C06.9	639	31.1	158	43.8	797	33.0

Salivary glands	C07.0-C08.9	36	1.8	16	4.4	52	2.2

Oropharynx	C09.0-C10.9	405	19.7	82	22.7	487	20.2

Nasopharynx	C11.0-C11.9	28	1.4	13	3.6	41	1.7

Hypopharynx	C12.0-C13.9	401	19.5	31	8.6	432	17.9

Oral cavity or pharynx unspecified	C14.0-C14.9	63	3.1	6	1.7	69	2.9

Nasal cavity/ear/paranasal sinuses	C30.0-C31.9	66	3.2	17	4.7	83	3.4

Larynx	C32.0-C32.9	479	23.3	50	13.9	529	21.9

The study will have adequate power to detect moderate increases in risk for exposures with a relatively low frequency. The minimum odds-ratios that may be detected with a 80% power at the 5% level of significance (one-tailed test) range from 1.2 (if the prevalence of exposure is 20%) to 1.7 (if the prevalence of exposure is 1%) for lung cancer, and from 1.2 to 1.8 for head and neck cancer. The number of subjects also allows to perform analyses among subgroups (e.g. by histologic type or subsite) with sufficient statistical power. For example, an analysis restricted to lung adenocarcinomas will have 80% power to detect odds-ratios greater than 1.4, 1.3 and 1.2 if the exposure prevalence is 5%, 10% and 20% respectively. In an analysis limited to oral cavity cancer, these figures become 1.5, 1.4 and 1.3.

## Discussion

With more than 2900 lung cancer cases, 2400 head and neck cancer cases and 3500 controls, ICARE is among the largest case-control studies worldwide on occupational risk factors for respiratory cancers, with an extensive and detailed collection of data on occupational exposures. The large number of subjects will allow to uncover exposures associated with low to moderate increase in risks, and to assess risks associated with infrequent and/or widely dispersed exposures.

Epidemiological analyses are ongoing. The first analyses concern associations with occupation and industry, and with exposures to asbestos, man-made mineral fibers, welding fumes and solvents.

From a public health perspective, it will be possible to estimate the proportion of respiratory cancers attributable to occupational exposures in France. Due to the lack of specific French data, only indirect estimates are currently available [34,35].

In addition to usual publications in scientific journals, special efforts will be devoted to the diffusion of the results in France, especially among occupational physicians, as well as chest and head and neck specialists. The findings of the study will provide a basis for a better understanding of the specific role of occupational carcinogens, and will contribute to inform health professionals on the issue of occupational cancer, in terms of prevention as well as compensation.

Furthermore, information on numerous risk factors other than occupational exposures is available, and many projects will rely on data from this study. For example, ICARE has provided recent and reliable data on the association between cigarette smoking and the risk of lung cancer in French women [36] and will be used to study social inequalities in respiratory cancers. The DNA biobank will allow to comprehensively investigate the role of genes, environmental factors and their interactions. A first ongoing project using data from the ICARE study aims to investigate complex interactions between well-established risk factors of three cancers among women (breast, lung and head and neck cancers) and inherited genetic variations, using a pathway candidate approach. A collection of tumor samples for cases included in ICARE is also planned, in particular to examine the relationships between tumor characteristics and environmental exposures. A first example of such studies is the comparison of molecular changes in sinonasal cancer with and without exposure to wood dust, which used data from ICARE [37]. ICARE is also included in the International Head and Neck Cancer Epidemiology (INHANCE) consortium [38] and in the International Lung Cancer Consortium (ILCCO) [39]. Participation to these consortia will further increase the number of research questions that can be addressed.

In conclusion, due to the population-based design, the quality of occupational data, the wide range of social, environmental and life-style factors, the biological collection, together with the large number of subjects, ICARE will improve our knowledge on occupational respiratory cancers and will provide an excellent framework for numerous studies in various fields.

## Competing interests

The authors declare that they have no competing interests.

## Authors' contributions

DL and IS are the joint PI's of the ICARE study. They conceived the study, designed the protocol, were in charge of the overall coordination of the study and wrote the manuscript. All authors read and approved the final manuscript.

## ICARE study group

Cancer registries: S. Bara (Manche), P. Bercelli (Vendée), A. Buemi (Haut-Rhin), M. Colonna (Isère), A. Danzon (Doubs), AV. Guizard (Calvados), F. Molinié (Loire-Atlantique), N. Raverdy (Somme), B. Trétarre (Hérault), M. Velten (Bas-Rhin);

INSERM UMRS 1018: S. Cénée, D. Cyr, A. Schmaus;

Département Santé travail, InVS: J. Févotte, C. Pilorget;

INSERM UMRS 775: C. Mulot, P. Laurent-Puig

## Pre-publication history

The pre-publication history for this paper can be accessed here:

http://www.biomedcentral.com/1471-2458/11/928/prepub
